# Real-life self-control conflicts in anorexia nervosa: An ecological momentary assessment investigation

**DOI:** 10.1192/j.eurpsy.2022.29

**Published:** 2022-06-16

**Authors:** Sophia Fürtjes, Maria Seidel, Stefan Diestel, Max Wolff, Joseph A. King, Inger Hellerhoff, Fabio Bernadoni, Katrin Gramatke, Thomas Goschke, Veit Roessner, Stefan Ehrlich

**Affiliations:** 1 Translational Developmental Neuroscience Section, Division of Psychological and Social Medicine and Developmental Neuroscience, Faculty of Medicine, Technische Universität Dresden, Dresden, Germany; 2 Department of Psychology, Technische Universität Dresden, Dresden, Germany; 3 Schumpeter School of Business and Economics, Faculty of Economy, University of Wuppertal, Wuppertal, Germany; 4 MIND Foundation, Berlin, Germany; 5 Department of Psychiatry and Psychotherapy, Charité Universitätsmedizin Berlin, Campus Charité Mitte, Berlin, Germany; 6 Eating Disorder Research and Treatment Center, Department of Child and Adolescent Psychiatry, Faculty of Medicine, Technische Universität Dresden, Dresden, Germany; 7 Department of Psychology, Technische Universität Dresden, Dresden, Germany

**Keywords:** Anorexia nervosa, eating disorders, ecological momentary assessment, self-control, self-control conflict

## Abstract

**Background:**

Individuals with anorexia nervosa (AN) are often thought to show heightened self-control and increased ability to inhibit desires. In addition to inhibitory self-control, antecedent-focused strategies (e.g., cognitive reconstrual—the re-evaluation of tempting situations) might contribute to disorder maintenance and enable disorder-typical, maladaptive behaviors.

**Methods:**

Over a period of 14 days, 40 acutely underweight young female patients with anorexia nervosa (AN) and 40 healthy control (HC) participants reported their affect and behavior in self-control situations via ecological momentary assessment during inpatient treatment (AN) and everyday life (HC). Data were analyzed via hierarchical analyses (linear and logistic modeling).

**Results:**

Conflict strength had a significantly lower impact on self-control success in AN compared to HC. While AN and HC did not generally differ in the number or strength of self-control conflicts or in the percentage of self-control success, AN reported self-controlled behavior to be less dependent on conflict strength.

**Conclusions:**

While patients with AN were not generally more successful at self-control, they appeared to resolve self-control conflicts more effectively. These findings suggest that the magnitude of self-control conflicts has comparatively little impact on individuals with AN, possibly due to the use of antecedent-focused strategies. If confirmed, cognitive-behavioral therapy might focus on and help patients to exploit these alternative self-control strategies in the battle against their illness.

## Introduction

Anorexia nervosa (AN) is characterized by extreme restriction of food intake and rigid behaviors that serve to control many aspects of daily life related to eating and weight gain [1, 2]. Individuals with AN are often thought to exercise excessive self-control to override food-related needs and desires in their relentless pursuit of thinness [3, 4]. However, little is known about how patients with AN might actually accomplish the high levels of control conceivably required to reach and maintain severely low bodyweight or how they experience self-control situations in everyday life.

Currently, influential theories propose that high self-control is achieved via effortful inhibition of prepotent behavioral impulses and resisting desires which interfere with personal goals [5]. Indeed, previous research has shown that inhibitory control is associated with less consumption of fatty foods and snacks [6–8], higher resistance to food desires, and successful weight loss [9]. Furthermore, a reduced activation in brain areas involved in inhibitory control (e.g., the right inferior frontal gyrus) has been shown to predict daily self-control failures, including food consumption [10, 11]. A plausible explanation for seemingly successful self-controlled behavior in AN might therefore be an unusually high capacity for inhibitory self-control, which enables resistance to temptations in the pursuit of disorder-typical goals (e.g., body/shape). This line of reasoning is supported by neuroimaging research suggesting that individuals with a history of AN show increased cognitive control (i.e., processes that organize behavior in a self-controlled goal-directed manner [12]) during reward processing [13], and that inhibitory control appears to require less effort in patients with AN [14–16]. Following this rationale, AN patients should have higher rates of success in overcoming conflicting self-control situations (i.e., resisting temptations such as watching TV instead of doing homework) in everyday life compared with healthy controls (HCs).

However, successful self-control might not be achieved solely via reactive resistance and inhibition of desires. Furthermore, these strategies may actually often be ineffective [17]. Behavioral strategies that proactively help to avoid tempting situations (e.g., environmental structuring or situation selection) or cognitive strategies such as, for example, goal priming (i.e., tempting cues are conditioned to activate self-control goals) and reconstrual (i.e., tempting cues are re-evaluated to be less desirable, similar to reappraisal processes) most likely also play a role in self-control by decreasing the necessity of effortful inhibition [18–22]. These so-called antecedent self-control strategies (especially reconstrual/reappraisal [20]), which have previously been mainly discussed in the context of emotion regulation [23], have recently gained interest [21]. Preliminary support suggesting that antecedent-focused self-control strategies are relevant to AN can be drawn from a study demonstrating that individuals with restrictive eating patterns experience less conflict when choosing the healthier of two food options (which might reflect cognitive processes such as reconstrual or goal priming [24]). If this can account for heightened self-control in AN patients, we would expect fewer and/or weaker self-control conflicts in everyday life relative to HC.

The present study investigated self-control processes in the everyday lives of patients with AN via ecological momentary assessment (EMA) [25], involving data acquisition via smartphone several times a day over a period of several days. By assessing the frequency of conflicting self-control situations, the strength of self-control conflicts and underlying desires, and the rate of successful self-control situations in patients with AN compared to HC, we aimed to elucidate actual self-control behavior in AN, outside of laboratory settings.

## Methods

### Participants

The initial sample consisted of 42 females with acute AN according to DSM-5 and 57 HCs (age range: 12.9–27.3 years). HCs were selectively recruited to minimize age differences between the two groups. To further optimize group comparisons and control for possible developmental effects, we implemented a pairwise matching algorithm [26]. The final sample consisted of 40 participants per group, which despite this procedure showed a small but significant age difference (Table 1). Diagnosis of AN was established using the expert form of the semi-structured interview for eating disorders (SIAB-EX) [27] and required a body mass index (BMI) below the 10th age percentile (if younger than 15.5 years) or below 17.5 kg/m² (if older than 15.5 years). AN patients were recruited within 96 h of admission to an inpatient treatment program at a university child and adolescent psychiatric department, which included individual, group, and family therapy. HCs had to be of normal weight, eumenorrheic, and without any history of psychiatric illness (assessed via the Mini International Neuropsychiatric Interview for Children and Adolescents [28]. All HCs were also assessed with the SIAB-EX and excluded if they showed any abnormal eating behavior. All participants were also interviewed with our own semi-structured interview to assess exclusion criteria (e.g., substance abuse and neurological conditions). The recruitment procedure was highly similar to our previous studies [29, 30] (see also the Supplementary Material [SM]).

**Table 1.** Sample characteristics and descriptive statistics.

	HC [*M* ± *SD*]	AN [*M* ± *SD*]	*t (p)*
Age	17.20 ± 2.79	15.90 ± 1.47	2.66^b^ (0.01)
BMI	21.17 ± 2.27	14.41 ± 1.45	15.89^b^ (0.00)
BMI-SDS	0.03 ± 0.63	−3.40 ± 1.10	16.87^b^ (0.00)
BDI-II	5.08 ± 5.58	26.05 ± 8.59	12.95^b^ (0.00)
EDI-2	132.84 ± 25.44	218.11 ± 34.52	12.58^b^ (0.00)
EMA			
Compliance	0.69 ± 0.18	0.82 ± 0.16	3.26^b^ (0.002)
Desires (total)	22.75 ± 15.17	24.30 ± 12.18	0.50 (0.62)
Desires per day (mean)	3.25 ± 2.17	3.47 ± 1.74	0.50 (0.62)
Desire strength (mean)	5.40 ± 0.57	5.72 ± 0.76	2.01^a^ (0.03)
Conflicts (total)	9.75 ± 7.06	14.10 ± 9.71	2.29^a^ (0.03)
Conflicting desires (%)	49 ± 26%	59 ± 27%	1.82 (0.07)
Conflict strength of conflicting desires (mean)	4.79 ± 0.99	4.52 ± 0.97	1.23 (0.22)
Resistance against conflicting desires (total)	7.63 ± 5.99	12.56 ± 9.30	2.80^b^ (0.01)
Resistance against conflicting desires (percentage)	76 ± 28%	82 ± 22%	0.97^a^ (0.05)
Success (total)	5.38 ± 4.12	8.45 ± 7.62	2.25^a^ (0.03)
Success in resisting all desires (%)	29 ± 20%	33 ± 24%	0.86 (0.39)
Success in resisting conflicting desires (%)	48 ± 31%	45 ± 30%	0.54 (0.33)
Success in resisting nonconflicting desires (%)	05 ± 0.8%	15 ± 22%	2.69^b^ (0.01)
Valence of affect (mean)	11.81 ± 1.26	7.47 ± 2.53	9.70^b^ (0.00)
Calmness (mean)	11.49 ± 1.56	8.15 ± 2.46	7.26^b^ (0.00)
Energetic arousal (mean)	9.13 ± 1.93	9.10 ± 1.52	0.08 (0.93)

*Notes: t* indicates values for independent group comparison. *n* = 40 per group. Age is given in years. Compliance is given in percentage of filled-out EMA questionnaires. All further variables are given as measured by the EMA questionnaire. Desire, binary variable [yes/no]; desire strength, continuous variable [1–7]; conflict, binary variable [yes/no]; conflict strength, continuous variable [1–7]; conflicting desires, desires for which conflict was affirmed; nonconflicting desires, desires for which no conflict was reported; resistance, binary variable [yes/no]; success, binary variable [yes/no]; valence of affect/calmness/energetic arousal, continuous variables [1–7].

Abbreviations: AN, anorexia nervosa; BMI, body mass index; BMI-SDS, body mass index standard deviation score; BDI-II, Beck Depression Inventory-II; BDI-SDS, body mass index standard deviation score; EDI-2, Eating Disorder Inventory-2; EMA, ecological momentary assessment, HC, healthy control.

a Significant at *α* ≤ 0.05.

b Significant at *α* ≤ 0.01.

### Materials and procedure

#### EMA assessment

**Figure 1.**
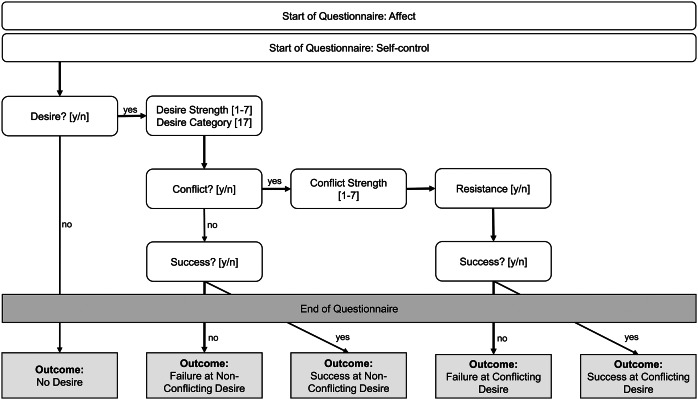
Procedure of the Ecological Momentary Assessment Questionnaire assessing self-control in real life and its outcomes.

Self-control was assessed via a short questionnaire based on the works of Hofmann et al. [31] and adapted from Wolff et al. [32]. An overview of the structure of the questionnaire and the assessed variables is visualized in Figure 1. At each prompt, participants were first asked whether or not they had experienced a situation in which they felt a *desire* to enact a certain behavior (since the last alarm) and also had the opportunity to do so (binary variable; yes/no). If this was the case, they were asked to select the desire category by choosing 1 out of 17 domains (e.g., eating, exercise, and watching TV; see the SM) and to indicate the *desire strength* on a scale from one (very weak) to seven (very strong). The next question assessed *conflict* (binary variable; yes/no) by asking whether or not participants had thought it would be better not to enact the desired behavior (i.e., if there was a reason to engage in self-control instead of simply enacting the desired behavior). Whenever participants reported a conflict, *conflict strength* was assessed on a scale from one (very weak) to seven (very strong) and participants were asked whether they had tried to resist the desire (*resistance,* binary variable; yes/no). Lastly, *success* (binary variable; yes/no) versus failure was assessed via the question whether participants had succeeded in not enacting the desired behavior.

Research has shown that self-control failure is often followed by negative affect in patients with AN, whereas controlled behavior may lead to positive affect. Alternative views exists as well, indicating that the relationship between failed self-control and affect in AN may be very nuanced [33]. Therefore, we also assessed affective states via a modified version of the Multidimensional Mood Questionnaire (MDMQ) [30, 34, 35]. Six items were adapted to assess *calmness*, *energetic arousal*, and *valence of affect* via visual analogue scales ranging from one to seven with opposite words as anchors (e.g., agitated calm).

#### Procedure

Before EMA, participants were interviewed, weighed, and measured. BMI and an age-adapted body mass index standard deviation score (BMI-SDS) [36, 37] were calculated. Participants also completed questionnaires to assess eating disorder (ED) symptoms (Eating Disorder Inventory-2 [EDI-2]) [38] and the depressive symptoms (Beck Depression Inventory-II [BDI-II]) [39]. Participants were given a detailed tutorial on how to handle the study smartphone and fill out the EMA questionnaires. Afterward, they received a study smartphone with the preinstalled app for data collection (xs.movisens) [40]. EMA assessment started the day following the tutorial. HCs completed the assessment for 7 days of their everyday life, and AN for 7 days of their daily routine during inpatient treatment. Participants were prompted eight times a day by an alarm to fill out the questionnaire. The alarms were semi-randomized during a 14-h period (individually adapted to fit different daily routines), allowing for data assessment over the course of the full day. After EMA assessment was completed, participants received monetary compensation in accordance with compliance (i.e., number of completed questionnaires). The study procedure was approved by the local ethics committee, and all participants (and the legal guardians of underage participants) gave written informed consent.

### Data analyses

The nested EMA data structure required hierarchical modeling with situations (Level 1) nested within subjects (Level 2). To analyze self-control, we performed a hierarchical generalized linear model (HGLM) via a population-averaged Bernoulli model for binominal outcome variables using the software HLM version 7 [41]. The population-averaged model was favored over the unit-specific model since we examined average differences in self-control success in two subpopulations (AN vs. HC) [42].

Success (i.e., not enacting the desired behavior, coded 1 for success and 0 for failure) was predicted by group, desire strength, conflict strength, and resistance. Because we tested cross-level moderation effects on random slopes, the Level-1 predictors’ desire strength and conflict strength were centered around the person’s mean to avoid biased estimations [43]. To further investigate whether the within-person relationship between these variables and self-control success differed between groups, the model also included cross-level interactions. Because previous research found that restrained eaters tend to experience less conflict regarding food decisions [24] and successful self-control might be promoted via cognitive strategies to reduce conflict [20], the conflict strength 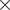 group interaction was our primary focus of interest. Therefore, a reduced model including only one cross-level interaction for conflict strength 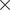 group is reported here; see the SM for results of a model including all cross-level interactions (Supplementary Table S8). Model comparisons via *χ*²-tests for differences in deviance confirmed that including additional cross-level interactions did not significantly improve model fit for any of the models (Supplementary Table S9).

Because we found that self-control success was less dependent on conflict strength in AN than HC, we asked whether the association between conflict and affective variables might be attenuated in AN. We therefore estimated further explorative linear hierarchical models in which valence of affect, calmness, and energetic arousal were (separately) predicted by the same variables described above, that is, group, desire strength and conflict strength (centered on the person’s mean), resistance, and success. Again, we report models including a cross-level interaction for conflict strength 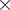 group in the main manuscript; for models with all cross-level interactions, see Supplementary Table S8.

Further control analyses were conducted adjusting for compliance rate with the EMA protocol, age, and BDI-II, excluding all situations with a desire in category “eating” and excluding AN participants of the binge-purge subtype. Sensitivity analyses within the AN group taking into account BMI-SDS and EDI-2 were also added (SM).

## Results

### Sample and descriptive statistics

Sample characteristics and descriptive statistics including group differences between AN and HC are reported in Table 1. As expected, AN had lower mean BMI and BMI-SDS, and higher ED symptom severity and depressive symptoms compared to HC. AN participants also showed higher compliance with the EMA protocol than HC.

As displayed in the second part of Table 1, there was no group difference in the number of reported *desires.* On average, AN experienced higher *desire strength* compared with HC. Although AN participants reported a higher total number of self-control *conflicts*, there was no group difference in the percentage of how often a conflict was affirmed when a desire was reported (*conflicting desires*) or in the mean reported *conflict strength.* In total, AN participants showed more *resistance* and experienced more situations with self-control *success.* There was no group difference in the percentage of self-control success overall as well as regarding *conflicting desires.* However, AN had a higher success rate in the case of *nonconflicting desires* (i.e., not enacting a desired behavior even though it did not stand in conflict with a superordinate goal).

Furthermore, as expected, AN reported more negative *valence of affect* and less *calmness* than HC (Table 1).

### Self-control in real life

**Figure 2.**
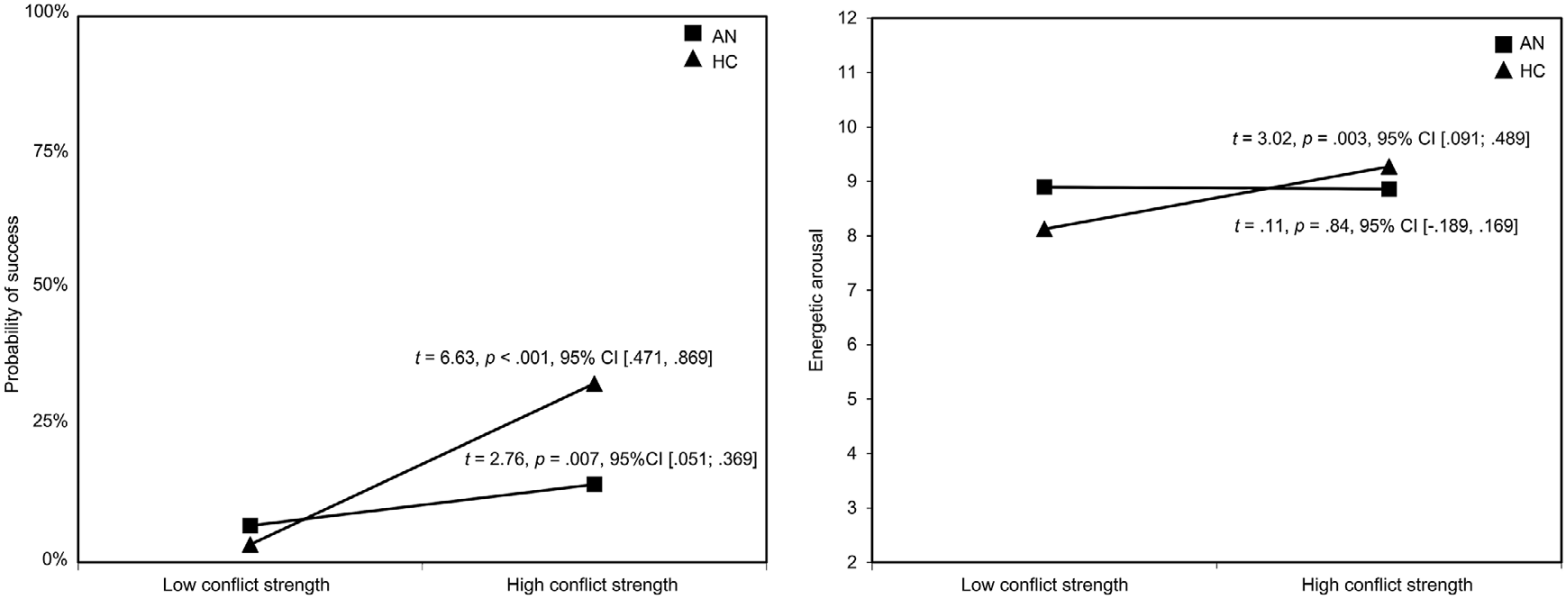
(A) Graphical representation of the positive relationship between conflict strength and self-control success, moderated by group (results from the hierarchical generalized linear model predicting success). (B) Graphical representation of the positive relationship between conflict strength and energetic arousal, moderated by group (results from the hierarchical linear model model predicting energetic arousal). *n* = 40 per group. AN, patients with Anorexia nervosa; HC, healthy control participants. Results of *t*-tests for significance of the slopes are shown for each group.

The results of the HGLM-based multilevel logistic regression showed that the probability of self-control success was generally higher when desires were weaker and conflict strength was stronger when resistance was exercised (Table 2). Overall, there was no significant group difference in the predicted probability of self-control success between AN and HC. The significant moderating effect of group on the relationship between conflict strength and success revealed that while higher conflict strength was generally associated with a higher probability of success, this association was weaker in AN, indicating that conflict strength is less relevant for success in AN than in HC (Figure 2A). Results remained robust when controlling for EMA compliance, age, and depressive symptoms (Supplementary Tables S1–S3). They also remained robust when excluding all situations with desires of the category “eating,” indicating that the found associations were not specific to ED-related self-control (Supplementary Table S4), and AN participants of the binge-purge subtype (Supplementary Table S5). Sensitivity analysis within the AN group showed no moderating effects of ED symptoms or BMI-SDS (Supplementary Tables S6 and S7).

**Table 2.** Effect of group and self-control variables on self-control success (HLGM) and affect (HLM).

	Success		Valence of affect	Calmness	Energetic arousal
	[*β*] (*p*)	OR [CI]	[*β*] (*p*)	[*β*] (*p*)	[*β*] (*p*)
Fixed effects					
Intercept	−2.13^b^ (<0.001)	0.12 [0.04; 0.36]	9.61^b^ (<0.001)	10.19^b^ (<0.001)	8.79^b^ (<0.001)
Situation level					
Trigger	0.00 (0.47)	1.00 [0.99; 1.01]	0.00 (0.94)	0.00 (0.48)	0.00 (0.70)
Desire strength	−0.54^b^ (<0.001)	0.59 [0.50; 0.68]	−0.09 (0.27)	−0.19^b^ (0.004)	−0.06 (0.48)
Conflict strength	0.44^b^ (<0.001)	1.55 [1.37; 1.76]	0.00 (0.99)	−0.10 (0.09)	0.14^a^ (0.04)
Resistance	2.05^b^ (<0.001)	7.77 [3.83; 15.77]	−0.19 (0.41)	−0.02 (0.94)	−0.14 (0.58)
Success			−0.25 (0.17)	−0.21 (0.25)	0.11 (0.57)
Person level					
Group	−0.08 (0.54)	0.92 [0.70; 1.21]	−2.23^b^ ( <0.001)	−1.73^b^ (<0.001)	0.09 (0.65)
Cross-level interaction					
Conflict strength × group	−0.23^b^ (<0.001)	0.80 [0.71; 0.90]	0.08 (0.24)	−0.01 (0.83)	−0.15^a^ (0.02)
Random effects					
*σ*²—residual variance at the situation level	0.21		4.77	4.04	4.83
*τ*—residual variance at the person level	1.53^b^ (<0.001)		3.24^b^ (<0.001)	4.06^b^ (<0.001)	2.64^b^ (<0.001)

*Notes:* Nonstandardized betas of the hierarchical analyses. *n* = 40 per group. Group was coded 1 (patient with Anorexia nervosa) and −1 (healthy control participant). All variables are given as measured by the EMA questionnaire. The model with self-control success as outcome is a hierarchical generalized linear model via a population-averaged Bernoulli Model for binary outcomes. Models with affect as outcome, which were part of an exploratory analysis, are hierarchical linear models. Desire strength and conflict strength were centered around the subject’s mean.

Abbreviations: EMA, ecological momentary assessment; OR, odds ratio.

a Significant at *α* ≤ 0.05.

b Significant at *α* ≤ 0.01.

### Self-control and affect (exploratory analyses)

Results of the exploratory multilevel logistic regression models showed that there was no significant association between self-control success and affect. Group had a significant effect on valence of affect and calmness, with AN experiencing less positive affect and less calmness than HC (Table 2). The cross-level interaction for conflict strength 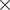 group had a significant effect on energetic arousal: in HC, higher conflict strength was associated with more energetic arousal—in AN, this relationship was not significant (Figure 2B and Table 2). Again, results remained robust when controlling for compliance, age, and depressive symptoms, when excluding all situations with a desire of the category “eating” (Supplementary Tables S1–S4), and AN participants of the binge-purge subtype (see Supplementary Table S5). Sensitivity analysis within the group of AN patients revealed that neither ED symptom severity nor BMI-SDS moderated the associations between conflict strength and energetic arousal (Supplementary Tables S6 and S7).

## Discussion

By analyzing momentary data collected over a period of 7 days in patients with acute AN and HC, we investigated how (elevated) self-control might be presented in and experienced by acutely underweight patients with AN in everyday life. We did not find significant group differences in either the frequency or strength of self-control conflicts. Furthermore, contrary to what one might expect, AN patients were not more successful at self-control in general, that is, the assumption that patients with AN are better at inhibiting unwanted impulses and resisting tempting desires was not supported by our findings. Instead, our findings suggest that a more nuanced look at self-control in daily life is necessary: while conflict strength played an important role for the probability of self-control success in HC (increased likelihood of success for stronger conflicts), this association was significantly less pronounced in AN. Pointing to the importance of antecedent self-control strategies [20, 21], patients with AN seem to act in a seemingly self-controlled manner even in situations of very low (or no) conflict strength.

As noted in the introduction, research has shown that successful self-control might be achieved not solely via inhibitory strategies, but also through antecedent-focused mechanisms, for example, by changing the conflict itself via goal priming or cognitive reconstrual [20, 21, 44]. Studies have shown that individuals who are successful at self-control show reduced conflict during choices between temptations versus goal-congruent options. They are quicker at resolving conflict, and possibly resolve conflict without strenuous effort by employing proactive self-control strategies [24, 45]. Previous research suggests that a less effortful resolution of self-control conflicts might be based on individual cognitive construals (i.e., subjective representations of events [46] that include a more abstract, higher-level perspective and subjective goals [46, 47]). These construals are thought to affect behavior, for example, by reducing the strength of temptations [21]. A speculative interpretation of our findings might be that AN patients achieve self-controlled behavior (e.g., not eating the tasty muffin) via reconstruals (e.g., representing negative attributes such as high caloric density when evaluating the situation, instead of positive attributes such as taste), which reduce the impact of conflict strength on success. Considering that our findings were not constricted to food temptations but included desires from many behavioral domains, this might be a domain-general self-control strategy in AN. A further indication that AN patients might use more antecedent-focused self-control strategies (such as reconstrual) than HC is reflected by our finding that higher conflict strength was associated with higher arousal only in HC, not AN. This might be due to the possibility that AN require less effort to resolve conflict (or report conflict strength that has already been modulated via, e.g., reconstrual), therefore not experiencing the arousal that could come along with more effortful conflict resolution in HC. The fact that AN also refrained from a desired behavior even though there was little or no conflict could possibly be explained by potent reconstruals which generally promote resistance to desires (e.g., restraint itself becomes a superordinate goal).

While our study was able to shed some light on self-controlled behavior of AN patients outside the laboratory, it should be noted that the interpretations outlined above are speculative and different explanations are possible. Previous research has shown that patients with acute AN often report anhedonia, avoidance of affect, and experiential avoidance [48–51]. They also often show increased levels of alexithymia, that is, difficulties in identifying and describing emotional states [52, 53], and decreased levels of interoception, that is, awareness of bodily signals [54]. Increased parasympathetic activity in the acute state of the disorder might also reduce the subjective experience of arousal [55, 56]. It could therefore be argued that we found conflict strength to be less relevant for self-control success in AN patients because, similarly to emotional states, self-control conflicts are experienced less intensely. However, sensitivity analyses revealed that BMI-SDS, which can be taken as a pathophysiological marker of the disorder, showed no associations with affect, desire strength, or strength conflict strength. We also found that AN reported stronger desires and more negative affect, but similar energetic arousal as HC (Table 1), which speaks against the aforementioned hypothesis. Another alternative interpretation could be that patients with AN experience self-control in itself as highly rewarding and therefore show self-controlled behavior independent from conflict strength. In line with the self-signaling theory, which proposes that people derive information about their identity from their behavior [57], AN patients may use self-control as a means to stabilize feelings of self-worth and may view self-discipline as an important aspect of their identity [58–60]. It is possible that these associations between self-control and self-worth or identity could explain our findings.

The findings we presented should be considered against the backdrop of some limitations. During the course of the study, the AN participants took part in comprehensive multimodal psychiatric and psychotherapeutic inpatient treatment program. Therefore, results might not reflect the daily life of individuals with AN outside of treatment. It should also be considered that, due to the restrictions of an inpatient setting, occurrence of desires as well as opportunities to act on them were likely reduced for AN participants. It is therefore possible that self-control conflicts could not have arisen in the same way for AN as for HC. However, we specifically assessed situations in which participants had a desire and also the opportunity to act on it. Of note, the groups did not differ significantly in the total number of reported desires or the percentage of conflict in situations with a desire (see Table 1). We cannot rule out the possibility that AN might experience more self-control conflicts outside of treatment, and therefore different results might be obtained if the study were to be repeated in an outpatient setting. A further aspect that should be noted regarding the different settings between the groups is the higher compliance in AN patients. It should also be kept in mind that our findings are based on momentary associations analyzed using regression-based statistical approaches. Therefore, no conclusions can be drawn regarding the causality between the associations between the self-control variables and affect. As stated in the method and results sections, the analyses with affect as an outcome variable were not part of our primary research question, but were added based on the findings of the analysis predicting self-control success. Future research should follow up on these exploratory findings in a hypothesis-driven investigation of associations between self-control behavior and affect. As previously mentioned, the relationship between successful versus failed self-control and affect in AN is complex [33], and the current investigation cannot do this topic justice. In addition, the present study did not investigate possible differences between AN participants with a restrictive versus binge-purge subtype, but merely confirmed the results in a purely restrictive subsample. Last but not least, our speculative interpretations regarding the possibility that AN patients make use of antecedent self-control strategies cannot be ascertained by our study design, which does not capture such processes. The reported conflict strength could possibly even reflect a value already modulated by such antecedent self-control strategies. Research using, for example, mouse-tracking paradigms [45, 61] to assess the dynamics of antecedent self-control processes is needed to investigate this question.

The research presented here indicates that patients with AN are not simply “better” at self-control than HC, but that they might resolve self-control conflicts more efficiently through strategies which lessen the impact of conflict strength on success or change the conflict itself. We speculated that this might be facilitated by antecedent-focused cognitive strategies such as goal priming or reconstrual, a possibility that should be addressed by future research. If patients with AN indeed show elevated self-control in daily life by resisting desires (even those with low conflict strength) through antecedent-focused cognitive strategies, cognitive-behavioral therapy might help patients to modify construals with the goal to re-evaluate food temptations as nonthreatening and food as enjoyable.

## Data Availability

The data that support the findings of this study are available from the corresponding author, S.E., upon reasonable request.

## Supplementary material

For supplementary material accompanying this paper visit https://doi.org/10.1192/j.eurpsy.2022.29. click here to view supplementary material
