# Supplementary material for: Real-life self-control conflicts in anorexia nervosa: An ecological momentary assessment investigation
Source: Eur Psychiatry. 2022 Jun 16;65(1):e39. doi: 10.1192/j.eurpsy.2022.29 (PMC9280923; doi:10.1192/j.eurpsy.2022.29)
Supplement: Supplementary file 1 [file S0924933822000293sup001.docx]

Fürtjes et al. (2022). Real-Life Self-Control Conflicts in Anorexia Nervosa

**Supplementary Material**

1. **Methods**

**1.1 Participants**

Information regarding exclusion criteria and possible confounding variables was obtained from all participants via the structured interview for anorexic and bulimic disorders (SIAB-EX; Fichter & Quadflieg, 2001), supplemented by our own semi-structured interview. Participants of the AN as well as the HC group were excluded if they had a lifetime history of any of the following clinical diagnoses: organic brain syndrome, schizophrenia, psychosis not otherwise specified, bipolar disorder, bulimia nervosa, or binge-eating disorder. Further exclusion criteria were: adipositas (BMI over 28 for 18 years and older or over the 94^th^ age percentile for participants younger than 18); IQ lower than 85; psychotropic medication within 4 weeks prior to study participation; current substance abuse; current inflammatory, neurologic, or metabolic illness; chronic medical or neurological illness that could affect appetite, eating behaviour, or body weight (e.g., diabetes); clinically relevant anaemia; pregnancy; breast feeding.

HC participants were recruited selectively with the goal to minimize age differences between the two groups. For further optimization of group comparison and control for possible developmental effects in the mostly juvenile sample, we implemented a pairwise matching based on the Munkres’ algorithm (Munkres, 1957) to minimize the mean pairwise distance. In the final sample, the mean age difference was 1.4 years between matched pairs and the age rage was 12.9 – 27.3.

**1.2 Ecological Momentary Assessment (EMA)**

Participants were provided with a study smartphone running the application which coordinated alarms, questionnaires, and answers. The study-procedure for the application was designed via an online platform (xs.movisens, Karlsruhe, Germany), which also managed data collection and immediate server upload for constant monitoring of compliance. Participants received a detailed tutorial on how to handle the smartphone, the application, and the content of the questionnaire. They were instructed to answer the questionnaire as soon as the alarm appeared, but were given an additional 30 minutes after the prompt when unable to reply (e.g., during class or work) or safety was a concern (e.g., while driving). Compensation was provided at the end of the study, in accordance with compliance rates. Data collection occurred via the signal-contingent assessment method: alarms occurred at eight semi-random times during a 14-hour period that was adapted for each individual to suit different daily routines. For AN participants, access to the study phone during inpatient treatment was ensured during the time of the assessments. Staff did not provide additional reminders to fill out the questionnaires.

The EMA questionnaire followed the structure described in the main manuscript, assessing affect, desire, conflict, resistance, and success. When a desire was reported, participants were asked to choose the category of the desire by selecting one of the following 17 options: eating, drinking (non-alcoholic), hygiene, exercise, sleep, laziness/“doing nothing”, being alone, being rude/doing something inappropriate, having company, intimacy/closeness, alcohol, smoking, internet, gaming, shopping, television, other.

Compensation for the EMA assessment was based on compliance to ensure high response rates: Participants received 0.30€ for each filled out questionnaire. If six or seven out of the eight daily alarms were answered, participants received 1.00€ bonus for this day. If all eight alarms were answered, the bonus increased to 2€. For the duration of the seven days participants could therefore receive a maximum of 33.00€.

1. **Additional Tables and Results**

To ensure that the results were not affected by possible confounding variables which could impact self-control and/or affect, the analyses reported in the main manuscript were repeated controlling for compliance, age, and depressive symptoms as measured by the BDI-II, and excluding all situations with a desire in the category “eating”. In addition, sensitivity analyses with success and energetic arousal as outcomes including severity of eating disorder (ED) symptoms as measured by the EDI-2 and BMI-SDS were conducted within the group of AN patients addressing the possible effects of ED specific variables on the findings presented in the main manuscript. Results are shown in *Table S1-S6* below.

As can be seen in the Tables below, all findings reported in the main manuscript remained robust in significance and direction of effects when controlling for compliance, age, and BDI-II. The sensitivity analyses within the group of AN patients showed that none of the clinical variables (EDI-2, BMI-SDS) had a significant direct impact on self-control success or energetic arousal. Findings also remained robust when excluding all situations with a desire in the category “eating”.

Because previous research found that restrained eaters tend to experience less conflict (Georgii, Schulte-Mecklenbeck, Richard, van Dyck, & Blechert, 2019) and successful self-control might be promoted via cognitive strategies to reduce conflict (Fujita, 2011), the conflict strength x group interaction was our primary focus of interest. Results for models including all possible cross-level interactions can be found below in *Table S7* and results of deviance χ²-tests for model comparison can be found in *Table S8*.

| *Table S1.* HGLM and HLM: effect of group and self-control variables on self-control success and affect – controlled for compliance | | | | |
| --- | --- | --- | --- | --- |
|  | **success** | **valence of affect** | **calmness** | **energetic arousal** |
| *Situation-Level:*  desire strength  conflict strength  resistance  success  *Person-Level:*  group  compliance  *Cross-Level Interaction:*  conflict strength x group | -.53**  .43**  1.97**  -.22  1.99**  -.22** | -.09  .00  -.19  -.26  --2.30**  1.15  .08 | -.19**  -.10  -.01  -.21  -1.81**  1.23  -.01 | -.06  .14*  -.13  .09  -.01  1.71  -.15* |
| *Notes.* Non-standardized betas of the hierarchical analyses. * = significant at α ≤ .01; ** = significant at α ≤ .01. *n* = 40 per group. Group was coded 1 = patient with Anorexia nervosa, -1 = healthy control participant. Compliance is given as percentage of filled out questionnaires. All other variables are given as measured by the EMA questionnaire. The model with self-control success as outcome is a hierarchical generalized linear model via a population-average Bernoulli-Model for binary outcomes. Models with affect as outcome are hierarchical linear models. Desire strength and conflict strength were centred around the subject’s mean. Trigger was included as a control variable on the situation-level in all analyses. | | | | |

| *Table S2.* HGLM and HLM: effect of group and self-control variables on self-control success and affect – controlled for age | | | | |
| --- | --- | --- | --- | --- |
|  | **success** | **valence of affect** | **calmness** | **energetic arousal** |
| *Situation-Level:*  desire strength  conflict strength  resistance  success    *Person-Level:*  group  age  *Cross-Level Interaction:*  conflict strength x group | -.53**  .42**  1.93**  -.12  -.04  -.21** | -.09  .00  -.19  -.25  -2.20**  .04  .08 | -.19**  -.10  -.02  -.21  -1.77**  -.06    -.01 | -.06  .14*  -.14  .10  .07  -.03  -.15* |
| *Notes.* Non-standardized betas of the hierarchical analyses. * = significant at α ≤ .01; ** = significant at α ≤ .01. *n* = 40 per group. Group was coded 1 = patient with Anorexia nervosa, -1 = healthy control participant. Age is given in years. All other variables are given as measured by the EMA questionnaire. The model with self-control success as outcome is a hierarchical generalized linear model via a population-average Bernoulli-Model for binary outcomes. Models with affect as outcome are hierarchical linear models. Desire strength and conflict strength were centred around the subject’s mean. Trigger was included as a control variable on the situation-level in all analyses. | | | | |

| *Table S3.* HGLM and HLM: effect of group and self-control variables on self-control success and affect – controlled for BDI-II | | | | |
| --- | --- | --- | --- | --- |
|  | **success** | **valence of affect** | **calmness** | **energetic arousal** |
| *Situation-Level:*  desire strength  conflict strength  resistance  success  *Person-Level:*  group  BDI-II total score    *Cross-Level Interaction:*  conflict strength x group | -.54**  .44**  2.04**  -.23  .01  -.22** | -.09  .00  -.20  -.25  -1.97**  -.02  .08 | -.19**  -.10  -.02  -.21  -1.41**  -.03  -.01 | -.06  .14*  -.15  .11  .69*  -.06*  -.15* |
| *Notes.* Non-standardized betas of the hierarchical analyses. * = significant at α ≤ .01; ** = significant at α ≤ .01. *n* = 40 per group. Group was coded 1 = patient with Anorexia nervosa, -1 = healthy control participant. BDI-II = Beck Depression Inventory. All other variables are given as measured by the EMA questionnaire. The model with self-control success as outcome is a hierarchical generalized linear model via a population-average Bernoulli-Model for binary outcomes. Models with affect as outcome are hierarchical linear models. Desire strength and conflict strength were centred around the subject’s mean. Trigger was included as a control variable on the situation-level in all analyses. | | | | |

| *Table S4.* HGLM and HLM: effect of group and self-control variables on self-control success and affect – excluding situations with desires in the category “eating” | | |
| --- | --- | --- |
|  | **success** | **energetic arousal** |
| *Situation-Level:*  desire strength  conflict strength  resistance  success  *Person-Level:*  group    *Cross-Level Interaction:*  conflict strength x group | -.52**  .41**  1.85**  -.07  -.23** | -.07  .13  -.03  .11  .19  -.14* |
| *Notes.* Non-standardized betas of the hierarchical analyses. * = significant at α ≤ .01; ** = significant at α ≤ .01. *n* = 40 per group. Group was coded 1 = patient with Anorexia nervosa, -1 = healthy control participant. All variables are given as measured by the EMA questionnaire. The model with self-control success as outcome is a hierarchical generalized linear model via a population-average Bernoulli-Model for binary outcomes. Models with affect as outcome are hierarchical linear models. Desire strength and conflict strength were centred around the subject’s mean. Trigger was included as a control variable on the situation-level in all analyses. | | |

| *Table S5*. Effect of group and self-control variables on self-control success (HLGM) and affect (HLM) – excluding AN participants of the binge-purge subtype | | |
| --- | --- | --- |
|  | **success** | **energetic arousal** |
| *Situation-Level:*  desire strength  conflict strength  resistance  success  *Person-Level:*  group    *Cross-Level Interaction:*  conflict strength x group | -.51**  .42**  2.22**  -.13  -.24** | -.06  .14*  -.14  .11  .15  -.15* |
| *Notes.* Non-standardized betas of the hierarchical analyses. OR = odds ratio. * = significant at α ≤ .05; ** = significant at α ≤ .01. *n* = 40 for HC, *n* = 32 for AN. Group was coded 1 = patient with Anorexia nervosa, -1 = healthy control participant. All variables are given as measured by the EMA questionnaire. The model with self-control success as outcome is a hierarchical generalized linear model via a population-average Bernoulli-Model for binary outcomes. Models with affect as outcome (which were part of an exploratory analysis) are hierarchical linear models. Desire strength and conflict strength were centred around the subject’s mean. Trigger was included as a control variable on the situation-level in all analyses. | | |

| *Table S6.* HGLM and HLM: effect of self-control variables on self-control success and affect in AN including EDI-2 | | |
| --- | --- | --- |
|  | **success** | **energetic arousal** |
| *Situation-Level:*  desire strength  conflict strength  resistance  success    *Person-Level:*  EDI-2 total score  *Cross-Level Interaction:*  conflict strength x EDI-2 | -.45**  .23**  1.76**  .01  .00 | .01  -.01  .23  -.03  -.01  .00 |
| *Notes.* Non-standardized betas of the hierarchical analyses. * = significant at α ≤ .01; ** = significant at α ≤ .01. *n* = 40. EDI-2 = Eating Disorder Inventory. All other variables are given as measured by the EMA questionnaire. The model with self-control success as outcome is a hierarchical generalized linear model via a population-average Bernoulli-Model for binary outcomes. Models with affect as outcome are hierarchical linear models. Desire strength and conflict strength were centred around the subject’s mean. EDI-2 was centred around the grand mean. Trigger was included as a control variable on the situation-level in all analyses. | | |

| *Table S7.* HGLM and HLM: effect of self-control variables on self-control success and affect in AN including BMI-SDS | | |
| --- | --- | --- |
|  | **success** | **energetic arousal** |
| *Situation-Level:*  desire strength  conflict strength  resistance  success    *Person-Level:*  BMI-SDS  *Cross-Level Interaction:*  conflict strength x BMI-SDS | -.45**  .26  1.76**  .09  .01 | .01  .20  .24  -.03  -.30  .07 |
| *Notes.* Non-standardized betas of the hierarchical analyses. * = significant at α ≤ .01; ** = significant at α ≤ .01. *n* = 40. BMI-SDS = body mass index standard deviation score. All other variables are given as measured by the EMA questionnaire. The model with self-control success as outcome is a hierarchical generalized linear model via a population-average Bernoulli-Model for binary outcomes. Models with affect as outcome are hierarchical linear models. Desire strength and conflict strength were centred around the subject’s mean. Trigger was included as a control variable on the situation-level in all analyses. | | |

| *Table S8.* HGLM and HLM: effect of group and self-control variables on self-control success and affect – including all possible cross-level interactions | | | | |
| --- | --- | --- | --- | --- |
|  | **success** | **valence of affect** | **calmness** | **energetic arousal** |
| *Situation-Level:*  desire strength  conflict strength  resistance  success    *Person-Level:*  group  *Cross-Level Interaction:*  desire strength x group  conflict strength x group  resistance x group  success x group | -.56**  .44**  2.00**  .17  .11  -.21**  -.29 | -.07  .00  -.21  -.25  -2.29**  -.10  .06  .07  .02 | -.16*  -.10  -.06  -.17  -1.88**  -.14*  -.03  .24  -.10 | -.07  .14*  -.17  .15  -.15  .07  -.16*  .41  -.17 |
| *Notes.* Non-standardized betas of the hierarchical analyses. * = significant at α ≤ .01; ** = significant at α ≤ .01. *n* = 40 per group. Group was coded 1 = patient with Anorexia nervosa, -1 = healthy control participant. All variables are given as measured by the EMA questionnaire. The model with self-control success as outcome is a hierarchical generalized linear model via a population-average Bernoulli-Model for binary outcomes. Models with affect as outcome are hierarchical linear models. Desire strength and conflict strength were centred around the subject’s mean. Trigger was included as a control variable on the situation-level in all analyses. | | | | |

| *Table S9*. Results of the deviance tests comparing model fit of the HGLM and HLM models with vs. without additional cross-level interactions | | | | | |
| --- | --- | --- | --- | --- | --- |
|  |  | **success** | **valence of affect** | **calmness** | **energetic arousal** |
| deviance of model with all cross-level interactions  deviance of model with one cross-level interaction  χ² | | 2671.4771  2673.4467  1.97 | 4293.50  4291.35  2.15 | 4162.43  4162.49  .06 | 4289.73  4289.81  .08 |
| *Notes:* Deviance of the hierarchical linear models. All cross-level interactions: group x desire strength, group x conflict strength, group x resistance. One cross-level interaction: group x conflict strength χ² = Chi-Test to compare fit of nested models; significant values indicate a better fit of the model with the lower deviance. None of the tests showed a significant difference in model-fit. *N* = 80. | | | | | |
